# RDX and miRNA Expression in B6C3F1 Mice

**DOI:** 10.1289/ehp.0800276

**Published:** 2009-03

**Authors:** Desmond I. Bannon, Mark Johnson, Larry Williams, Valerie Adams, Edward Perkins, Kurt Gust, Ping Gong

**Affiliations:** U.S. Army Center for Health Promotion and Preventive Medicine, Directorate of Toxicology, Aberdeen Proving Ground, Maryland, E-mail: desmond.bannon@us.army.mil; Environmental Laboratory, U.S. Army Engineer Research and Development Center, Vicksburg, Mississippi

In a recent issue of *EHP* Zhang and Pan (2009) reported on the effects of the explosive hexahydro-1,3,5-trinitro-1,3,5-triazine (RDX) on the differential expression of microRNAs (miRNAs) in brain and liver of B6C3F1 mice. It is always of interest when new technologies are applied to existing toxi cologic problems, with a view to increasing our understanding of the effect on, or risk to, humans. However, in the abstract of their article originally published online (but deleted from the final version), Zhang and Pan (2009) concluded that “environmental toxicant exposure alters the expression of a suite of miRNAs that in turn regulates gene expression which may lead to carcinogenesis, developmental, neuronal, and reproductive toxicity”; they reached this conclusion in the absence of any observations for dose response, clinical chemistry, histopathology, or neurotoxicity. Because their results do not support these conclusions, we felt a response was warranted.

Zhang and Pan (2009) exposed B6C3F1 female mice to RDX in food. The mouse chow was sprayed with a solution of acetone-dissolved RDX and allowed to dry; this resulted in a formulation chow containing 5 mg RDX/kg of food. At this dose of RDX, we estimated that the mice received approximately 0.75–1.5 mg/kg body weight/day, based on mouse food consumption of 3–6 g/ day and an average body weight of 20 g. To put this dose in perspective, the 2-year cancer study on which RDX risk assessment was based (Lish et al. 1984) used oral doses of 0, 1.5, 7.0, 35, and 175 mg RDX/kg/day in the same mouse strain, with statistically significant liver cancers found only in the 35-mg/ kg dose group. The dose used by Zhang and Pan in their 1-month study was therefore less than the lowest dose in the 2-year mouse cancer study and over 20 times lower than the only dose of RDX associated with cancer. Furthermore, given that only a fraction of the exposed animals developed cancer at the 35-mg/kg dose in the 2-year study (Lish et al. 1984), we wonder how let-7 and other miRNAs used by Zhang and Pan (2009) identify which animals could potentially get cancer at a higher dose (i.e. susceptibility), or whether all animals could develop cancer even at this low dose (i.e., over prediction).

At high oral exposures, RDX causes tonic–clonic seizure, an effect that has been well correlated with internal dose (blood RDX was not measured in Zhang and Pan’s study). The mode of action of RDX is thought to be direct because seizures can occur within minutes of dosing. Zhang and Pan (2009) reported that brain derived miRNA 206 was increased 26-fold and brain-derived neurotrophic factor (BDNF) was computationally identified as a downstream target, with the direction of change presumably inhibitory on BDNF. Current literature shows that BDNF is actually up-regulated in response to seizure-inducing agents, such as kainite (Revuelta et al. 2005) and domoic acid (Doucette et al. 2004). Whether other presumed targets of miRNA would be up-regulated is not known, making verification of miRNA targets (mRNA) critical in the validation of this kind of study.

Although miRNAs have been used extensively to examine the profiles of small RNAs in distinct phenotypes such as cancer, their significance as predictors of toxic insult or disease has not been demonstrated. The field of miRNAs is burgeoning with publications (1,738 in 2008), many of which involve the retro spective examination of diseased tissue (tumors) for changes in the expression of miRNA species. Prospective work relating chemical exposure to changes in miRNA as predictors of imminent disease has been less successful, and a study of dioxin found miRNAs refractive (Moffat et al. 2007). More important, some reviews (Kozak 2008) caution against overinterpretation of miRNA data, especially without verification of downstream targets.

It has been said that “a difference, to be a difference, should make a difference.” We found it difficult to assess the biological significance of the suite of differentially regulated miRNAs and their computational targets culled from the study of Zhang and Pan (2009); although these miRNAs could be associated with exposure to RDX, they do not seem related to disease. In our opinion, Zhang and Pan’s results fall short of their experimental hypothesis that exposure to specific environmental agents, such as RDX, would cause alteration in miRNA expression and that “the altered miRNA expression contributes to carcinogenesis.” For innovative work of this kind, a solid model of exposure–disease is always a good starting point, coupled with the classical toxicology stalwarts of dose response and positive/negative controls, and of course, verification of putative targets. Here, we feel that poor study design, absence of phenotype, and overinterpreation of data significantly weakened a potentially informative body of work.
